# Knowledge, attitudes, and practices (KAP) toward seasonal influenza vaccine among college students under the COVID‐19 pandemic in South China

**DOI:** 10.1002/iid3.1110

**Published:** 2023-12-14

**Authors:** Di Wu, Yuexue Mai, Pan Liu, Jie Long, Qun Liu, Tiantian Wu, Dedong Wang, Xiangzhi Hu, Weiquan Lin, Xuejiao Chen, Zhoubin Zhang, Pengzhe Qin

**Affiliations:** ^1^ Department of Biostatistics and Cancer Registration Guangzhou Center for Disease Control and Prevention Guangzhou China; ^2^ School of Public Health, Institute of Public Health Guangzhou Medical University & Guangzhou Center for Disease Control and Prevention Guangzhou China; ^3^ Department of Plastic Surgery, Medical School, The Second Affiliated Hospital Zhejiang University Hangzhou China; ^4^ Office of Educational Administration Guangzhou Medical University Guangzhou China; ^5^ Affiliated Cancer Hospital Guangzhou Medical University Guangzhou China; ^6^ Guangdong Provincial Institute of Biological Products and Materia Medica Guangzhou China; ^7^ Health and Quarantine Laboratory Guangzhou Customs District Technology Centre Guangzhou China; ^8^ Department of Public Health and Preventive Medicine, School of Medicine Jinan University Guangzhou China; ^9^ Office of Academic Research, Guangdong Provincial People's Hospital (Guangdong Academy of Medical Sciences) Southern Medical University Guangzhou China

**Keywords:** coronavirus disease 2019 (COVID‐19), influenza, knowledge, attitudes and practices (KAP), vaccine

## Abstract

**Background:**

The control measures of the coronavirus disease 2019 (COVID‐19) pandemic had influenced the activity of the influenza virus, and we were wondering the knowledge, attitudes, and practices (KAP) toward seasonal influenza vaccine among college students were having at the context of the COVID‐19 pandemic.

**Methods:**

Online questionnaire survey of the college students was conducted and the data was collected anonymously, cross‐sectional study were used to describe the distribution of the KAP.

**Results:**

There were 815 respondents in our study. Most participants have a high recognition of the effectiveness and safety of the influenza vaccine. However, a low awareness rate of influenza vaccine knowledge and vaccination rate (18%) against influenza was observed among college students. The education level and major would have a higher weight on the influence of KAP among the college students. Binary logistic regression analysis showed that the sex (OR = 2.163, *p* < .001), age (OR = 2.242, *p* < .001), heard of the influenza vaccine (OR = 2.655, *p* = .014), and “How necessary do you think vaccinating every year is?” (OR = 3.947, *p* < .001) of the college students were the main factors that affect the KAP on influenza vaccination.

**Conclusions:**

Our study provided an insight into the KAP of influenza vaccine among college students in South China. The vaccination rate and acceptability of influenza vaccine in college students are higher than that in the whole population.

## 1 BACKGROUND

Seasonal influenza caused by influenza viruses (IVs) infection speared all over the world which has a serious impact on the global health.^1^ The IVs can be transmitted through respiratory droplets, aerosol, and close contact with infected individuals and may trigger a series of severe illness and even lead to death, studies have proved that the vaccine can effectively decrease the risk of infection, reduce the severity, and the risk of death after infection.^1^, ^2^, ^3^, ^4^ Nowadays, the pandemic of severe acute respiratory syndrome coronavirus 2 (SARS‐CoV‐2) and the control measures of coronavirus disease 2019 (COVID‐19) had a strong impact on the epidemic of IVs.^5^, ^6^, ^7^, ^8^

However, vaccination coverage remains low in developing countries,^3^ which is far lower than 75% of the target populations recommended by World Health Organization (WHO).^9^ It's critical that herd immunity protects vulnerable populations and controls the pandemic.^10^ Vaccine hesitation is one of the most obstacles contributing to vaccine uptake and achievement of herd immunity,^9^ vaccination hesitation is to refuse or postpone vaccination which is a worldwide phenomenon that undermines global public health.^11^ The reasons for WHO's vaccine hesitation are classified into three aspects: confidence, complacency, and convenience, namely the “3Cs” model: confidence refers to the confidence of vaccinators in the safety and effectiveness of vaccines, health system and vaccinators, and decision‐makers, complacency refers to the psychology that people think that the risk of vaccine‐preventable diseases is low, and ignores the necessity of vaccination, convenience refers to the accessibility of vaccination, the acceptability of price, and so forth.^12^ It may be influenced by education levels, income, age, and so on.^9^, ^13^

College students are a unique demographic group, which are well‐educated, and the group had a crowded living environment and study places, what's more, frequent social activities are the typical characteristics of the population, which all lead to a higher risk of infection of IVs.^14^ The outbreak of influenza in a campus would undermine the regular teaching activities and students' academic performance. Furthermore, college students are one of the most active populations, in terms of social activities which may cause widespread of influenza.^15^ Understanding the willingness of influenza vaccination among college students is crucial for influenza prevention and control. There are few studies published on Chinese college students' vaccine hesitancy, especially during the pandemic of COVID‐19. Moreover, there were few surveys on the college students' vaccine hesitation founded and could be referred for the existing twindemic of influenza and COVID‐19.^16^

As a result, we conducted a survey among college students in Guangzhou, China, including knowledge, attitudes, and practices (KAP),^17^ to investigate the willingness of college students to hesitate and resist the possible factors in the current vaccination process, and to focus on college students in the current vaccination process to prepare for the expansion of immunization coverage in China, and to give better evidence of effective vaccination strategies in China.

## 2 METHODS

### 2.1 Participant selection

We conducted a cross‐sectional survey during January 2021. The participants of this study need to meet the conditions as follows^1^: University Student in Guangzhou^2^; volunteering to participate in the study^3^; using a smartphone to fill the questionnaire. And the exclusion criteria^1^: Unfinished questionnaire^2^; fill in the questionnaire in less than 2 min or more than 10 min.

### 2.2 Data collection and quality management

Ethical and anonymous data use statements were declared at the first beginning of the questionnaire and agreed by the expectants. To ensure the validity of the questionnaire, a pretest was carried out. Finally, the questionnaire consisted of four parts, including^1^: demographic data^2^; knowledge of the influenza vaccine^3^; attitude toward the influenza vaccine^4^; influenza vaccine practices. Closed‐ended or multiple‐choice were utilized in all questions.

### 2.3 Knowledge

There are three questions about the knowledge. The first one is “What population needs the influenza vaccine?.” And the answer consisted of five choices, including: Children (6 months to 5 years old); elderly people (>60 years); chronic disease patients; medical staff; and those who work with babies and children. The second is “When are recommended for influenza vaccination?.” The answer choices are as follows: January to March; April to June; July to September; October to December; do not know. The third is “How necessary do you think vaccinating every year is?” The answers were: necessary, not necessary, and do not know. The first two topics are multiple‐choice questions, and the last one is a single‐choice question.

### 2.4 Attitudes

The participants' attitudes toward the influenza were made up of five questions. Including^1^: Do you worry about getting influenza?,^2^ Have you ever heard of the influenza vaccine?,^3^ Do you think the influenza vaccine can protect you from getting influenza?,^4^ How safe do you think the influenza vaccine is?,^4^ and Will you advise your family and friends to be vaccinated with the influenza vaccine? All details were shown in Table 2.

### 2.5 Practices

The questions following were utilized to evaluate the practices toward the influenza vaccine in the study. The first question was “Have you been vaccinated with the influenza vaccine within the past 4 years?” and the answers were “Yes” or “No” While the participant answered “Yes” you need to report the frequency of influenza vaccination, the approach that led you to be vaccinated with the influenza vaccine and continue willingness to vaccinate. When the participants indicated their willingness to vaccinate or not and then reasons need to report.

### 2.6 Data analysis

Excel was utilized to collect the data and put it in order. SPSS 18.0 was used to analyze the data. The *χ* ^2^ test and/or Fisher's exact test were used to compare the proportions of groups with different ages, family incomes, and educational levels. To make the relationships between willingness to knowledge, attitude, and practice sense, the multivariate logistic was used. The odds ratios (ORs) were utilized to confirm the independent prediction factor.

## 3 RESULTS

### 3.1 Demographics

There is a total of 815 college students participated in the survey, and 815 questionnaires data are valid. The male (*N* = 358, 43.9%) participated in the survey are less than female (*N* = 457, 56.1%). The median age of participants is 20. And the group of age 18−20 years old is the main group (*N* = 359, 44.1%). Most of the involved participants are in their first‐year college (*N* = 413, 50.7%). Medicine is the main major of these college students (*N* = 566, 69.5%), the major ranked second is engineer (*N* = 194, 23.8%), and the last is science or other (*N* = 55, 6.8%). Per capita monthly income in family ranged from <1000 to 50,001 Yuan. Most of their income are less than 10,000 Yuan. Moreover, the overall result can be seen in Table 1.

**Table 1:** Demographic characteristics of the study participants in Ghuangzhou (*N* = 815).

Demographic information	*n*	% (*n*/*N* × 100%)	Coverage (*a*/*n* × 100%)
Sex			
Male	358	43.9	25.7 (92/358)
Female	457	56.1	12.0 (55/457)
Age (years)			
<18	9	1.1	22.2 (2/9)
18−20	359	44.1	24.5 (88/359)
20−22	312	38.3	13.8 (43/312)
22−24	93	11.4	8.6 (8/93)
24−26	24	2.9	20.8 (5/24)
>26	18	2.2	5.6 (1/18)
Education			
First‐year college	413	50.7	26.2 (108/413)
Second‐year college	91	11.2	13.2 (12/91)
Third‐year college	172	21.1	8.1 (14/172)
Fourth‐year college	74	9.1	9.5 (7/74)
Fifth‐year college	20	2.5	5.0 (1/20)
Master students	45	5.5	11.1 (5/45)
Major			
Medicine	566	69.5	14.8 (84/566)
Engineering	194	23.8	27.3 (53/194)
Science and others	55	6.8	18.2 (10/55)
Per capita monthly income in family			
<1000	85	10.4	16.5 (14/85)
1001−3000	298	36.6	19.1 (57/298)
3001−6000	226	27.7	17.3 (39/226)
6001−10,000	118	14.5	17.8 (21/118)
10,001−20,000	57	7.0	19.3 (11/57)
20,001−50,000	20	2.5	10.0 (2/20)
50,001	11	1.4	27.3 (3/11)

*Note*: *N* represents the number of participants in this survey, *n* represents the number of participants for each demographic group, *a* represents the number of participants who received the seasonal influenza vaccination within the last 4 years. Coverage describes the vaccination rate over the last 4 years (coverage = *n*/*N* × 100%).

### 3.2 Knowledge

In this study, the awareness rate of influenza vaccine knowledge among college students was low. Most of the participants deem children (*N* = 689, 85.6%) and elderly people (*N* = 650, 79.8%) were influenza vaccine needing populations. And followed by medical staff (*N* = 567, 69.6%), those who worked with babies and children (*N* = 543, 66.6%), and chronic disease patients (*N* = 430, 52.8%). However, only a few participants knew January to March (*N* = 253, 31%) and October to December (*N* = 153, 18.8%) were the vaccination‐recommended seasons. There was nearly one‐third of participants thought that influenza vaccination was not necessary (*N* = 262, 32.1%). Unabridged data were shown in Table 2. In the group analysis, the results showed that the group which age >20 years old or had a master's degree may have greater knowledge of recommended population for influenza vaccination (*p* < .05). About the recommended season for influenza, the different groups of age, education, and major both showed significant differences. And more details were shown in Table 3.

**Table 2:** Knowledge of the influenza vaccine among the study participants in Guangzhou (*N* = 815).

Knowledge	*n*	% (*n*/*N* × 100%)	95% CI lower	95% CI upper
What population needs the influenza vaccine?				
Children (6 months to 5 years old)	698	85.6	83.4	87.9
Elderly people (>60 years)	650	79.8	76.9	82.5
Chronic disease patients	430	52.8	49.2	56.3
Medical staff	567	69.6	66.4	72.6
Those who work with baby and children	543	66.6	63.2	69.9
What seasons are recommended for influenza vaccination?				
January to March	253	31.0	28.0	34.4
April to June	133	16.3	13.6	18.8
July to September	122	15.0	12.5	17.4
October to December	153	18.8	16.1	21.3
Do not know	154	18.9	16.2	21.8
How necessary do you think vaccinating every year is?				
Necessary	362	44.4	41.2	47.6
Not necessary	262	32.1	29.1	35.3
Do not know	191	23.4	20.5	26.4

**Table 3:** Knowledge of the influenza vaccine in the age, education, major, and income group analysis (*N* = 815).

	<20 (*n* = 368)	≥20 (*n* = 447)	*χ* ^2^	*p*	Undergraduate students (*n* = 770)	Master students (*n* = 45)	*χ* ^2^	*p*	Medicine (*n* = 566)	Engineering (*n* = 194)	Science and others (*n* = 55)	*χ* ^2^	*p*	<3000 (*n* = 383)	3000−10,000 (*n* = 344)	>10,000 (*n* = 88)	*χ* ^2^	*p*
Knowledge																		
What population needs the influenza vaccine?																		
Children (6 months to 5 years old)	307	391	2.690	.101	659	39	0.841	.041	488	161	49	1.795	.408	336	289	73	2.617	.270
Elderly people (>60 years)	296	354	0.192	.661	612	38	0.649	.421	447	160	43	1.186	.553	311	274	65	2.390	.303
Chronic disease patients	177	253	5.853	.016	400	30	3.695	.055	310	93	27	3.025	.220	207	182	41	1.601	.449
Medical staff	238	329	7.599	.006	534	33	0.319	.572	404	127	36	2.859	.239	261	245	61	0.812	.666
Those who work with baby and children	233	310	3.307	.069	513	30	<0.001 44	.995	387	123	33	2.771	.250	263	228	52	2.984	.225
What seasons are recommended for influenza vaccination?																		
January to March	121	132	14.872	.005	239	14	16.761	.002	176	62	15	18.095	.021	127	99	27	12.650	.124
April to June	68	65			130	3			84	43	6			61	61	11		
July to September	43	79			116	6			92	17	13			47	65	10		
October to December	56	97			135	18			112	29	12			68	64	21		
Do not know	80	74			150	4			102	43	9			80	55	19		
How necessary do you think vaccinating every year is?																		
Necessary	152	210	2.634	.268	340	22	0.886	.642	263	80	19	5.068	.280	172	144	46	5.630	.229
Not necessary	125	137			247	15			173	70	19			120	122	20		
Do not know	91	100			183	8			130	44	17			91	78	22		

### 3.3 Attitudes

In terms of attitudes, most participants have a high recognition of the effectiveness and safety of the influenza vaccine. All related data are shown in Table 4.

**Table 4:** Attitudes toward the influenza vaccine among the study participants in Guangzhou (*N* = 815).

Attitude	*n*	% (*n*/*N* × 100%)	95% CI lower	95% CI upper
Do you worry about getting influenza?				
Very worried	122	15.0	12.8	17.5
Worried	513	62.9	59.5	66.1
Not worried	180	22.1	19.3	25.3
Have you ever heard of the influenza vaccine?				
Yes	694	85.2	82.8	87.5
No	121	14.8	12.5	17.2
Do you think the influenza vaccine can protect you from getting influenza?				
Yes	664	81.5	78.7	84.0
No	49	6.0	4.4	7.6
Do not know	102	12.5	10.3	14.8
How safe do you think the influenza vaccine is?				
It is safe and with no side effects.	112	13.7	11.4	16.2
It is basically safe and with some side effects.	692	84.9	82.5	87.4
It is not safe and with obvious side effect.	11	1.3	0.6	2.2
Will you advise your family and friends to be vaccinated with the influenza vaccine?				
Yes	659	80.9	78.2	83.4
No	156	19.1	16.6	21.8

In the group analysis, the different groups may have a different attitude. The different groups of age, education, and income have significant differences (*p* < .05). The results showed that higher education degree and the elder may relate to low concern about influenza infection. Then, about advising family or friends to get the vaccine or not, the group age, education, and major have significant differences (*p* < .05). The results are shown in Table 5.

**Table 5:** Attitudes toward the influenza vaccine in the age, education, major, and income group analysis (*N* = 815).

	<20 (*n* = 368)	≥20 (*n* = 447)	*χ* ^2^	*p*	Undergraduate students (*n* = 770)	Master students (*n* = 45)	*χ* ^2^	*p*	Medicine (*n* = 566)	Engineering (*n* = 194)	Science and others (*n* = 55)	*χ* ^2^	*p*	<3000 (*n* = 383)	3000−10,000 (*n* = 344)	>10,000 (*n* = 88)	*χ* ^2^	*p*
Attitude																		
Do you worry about getting influenza?																		
Very Worried	63	59	9.458	.009	120	2	12.968	.002	86	28	8	9.108	.058	65	48	9	10.008	.040
Worried	241	272			489	24			345	137	31			250	210	53		
Not Worried	64	116			161	19			135	29	16			68	86	26		
Have you ever heard of the influenza vaccine?																		
Yes	315	319	0.105	.746	655	39	0.086	.769	474	172	48	2.969	.227	328	289	77	0.810	.667
No	53	68			115	6			92	22	7			55	55	11		
Do you think the influenza vaccine can protect you from getting influenza?																		
Yes	300	364	1.889	.389	626	38	0.342	.852	458	159	47	1.024	.906	302	288	74	3.413	.491
No	26	23			47	2			35	12	2			27	18	4		
Do not know	42	60			97	5			73	23	6			54	38	10		
How safe do you think the influenza vaccine is?																		
It is safe and with no side effects.	49	63	1.606	.448	105	7	1.112	.488	74	33	5	5.667	.194	52	49	11	0.450	.978
It is basically safe and with some side effects.	312	380			655	37			484	160	48			325	291	76		
It is not safe and with obvious side effects.	7	4			10	1			8	1	2			6	4	1		
Will you advise your family and friends to be vaccinated with the influenza vaccine?																		
Yes	319	340	14.714	<.0011	629	30	6.198	.013	443	172	44	10.108	.006	317	275	67	2.357	.308
No	49	107			141	15			123	22	11			66	69	21		

### 3.4 Practice

Table 6 lists the results of the participants' practice. Merely 18% of participants have been vaccinated within the past 4 years. In the group of people vaccinated before, we found that there is a small number of participants who have an annual vaccination (*N* = 44, 5.5%). Doctors' recommendation is the most approach that led to being vaccinated (*N* = 74, 9.1%). And the people who were vaccinated before will have a high willingness to vaccinate again in the future (*N* = 104, 70%). The main obstacle to not continuing to vaccinate is limited vaccination sites (*N* = 76, 9.3%).

**Table 6:** Practices toward the influenza vaccine among the study participants in Guangzhou (*N* = 815).

Practices	*n*	% (*n*/*N* × 100%)	95% CI lower	95% CI upper
Have you been vaccinated with the influenza vaccine within the past 4 years?				
Yes	147	18.0	15.2	20.7
No	668	82.0	79.3	84.8
The frequency of influenza vaccination				
Every year	44	5.4	3.9	7.0
Every 2−3 years	56	6.9	5.2	8.7
Occasionally	47	5.8	4.3	7.4
The approach that led you to be vaccinated with the influenza vaccine				
Advised by doctor	74	9.1	7.1	11.2
Voluntarily vaccinated	49	6.0	4.3	7.7
Recommended by family members or friends	17	2.1	1.2	2.9
Others	7	0.9	0.2	1.6
Willingness to continue vaccinating				
Yes	103	12.6	10.2	15.0
No	11	1.3	0.6	2.1
Depends	33	4.0	2.8	5.4
The reason you will not to continue to vaccinate				
High cost	41	5.0	3.6	6.6
Limited effect	50	6.1	4.5	7.9
Side effects and poor safety	29	3.6	2.5	4.9
Limited vaccination sites	76	9.3	7.4	11.2
Others	8	1.0	0.4	1.7
The reason you did not vaccinate				
High cost	52	6.4	4.7	8.1
In good health. The influenza is not a serious illness	213	26.1	23.1	29.2
Side effects and poor safety	82	10.1	8.0	12.0
Limited effect	38	4.7	3.3	6.1
Unknown vaccination sites	253	31.0	28.0	34.4
No time	143	17.5	15.1	20.5
Never heard of it	189	23.2	20.2	26.1
Vaccine shortage	29	3.6	2.3	4.9
Contraindications	8	1.0	0.4	1.7
Vaccination sites is far	47	5.8	4.3	7.5
Others	26	3.2	2.1	4.5
Do you intend to receive influenza vaccinate in the future?				
Yes	89	10.9	8.8	13.4
No	357	43.8	40.2	47.6
It depends	222	27.2	24.4	30.4

In the group of people who did not vaccine within the past years, the majority of reasons for did not vaccinate are unknown vaccination sites (*N* = 253, 31%). Contrary to the people who were vaccinated before, most of them do not want to vaccinate the influenza vaccine in the future (*N* = 35.7%, 43.8%).

As the group analysis results are shown in Table 7, the different groups of age or major may influent the vaccination decision (*p* < .05). The elder (age >20) had a lower vaccination rate while the group of medicine majors had more vaccination. Higher‐income groups (>10,000) may have more concerned about the limited effect of the vaccine (*p* < .05). The group of engineering major showed high willingness to vaccinate in the future (*p* < .05).

**Table 7:** Practices toward the influenza vaccine in the age, education, major, and income group analysis (*N* = 815).

	<20 (*n* = 368)	≥20 (*n* = 447)	*χ* ^2^	*p*	Undergraduate students (*n* = 770)	Master students (*n* = 45)	*χ* ^2^	*p*	Medicine (*n* = 566)	Engineering (*n* = 194)	Science and others (*n* = 55)	*χ* ^2^	*p*	<3000 (*n* = 383)	3000−10,000 (*n* = 344)	>10,000 (*n* = 88)	*χ* ^2^	*p*
Practice																		
Have you been vaccinated with the influenza vaccine within the past 4 years?																		
Yes	90	57	18.705	<.001	142	5	1.545	.214	84	53	10	15.219	<.001	71	60	16	0.149	.928
No	278	390			628	40			482	141	45			312	284	72		
The frequency of influenza vaccination																		
Every year	28	16	0.635	.728	41	3	5.192	.075	28	12	4	4.353	.360	22	18	4	0.541	.969
Every 2−3 years	32	24			56	0			34	19	3			28	22	6		
Occasionally	30	17			45	2			22	22	3			21	20	6		
The approach that led you to be vaccinated with the influenza vaccine																		
Advised by doctor	46	28	2.917	.405	72	2	1.322	.752	41	28	5	8.381	.172	34	33	7	7.183	.270
Voluntarily vaccinated	27	22			47	2			27	18	4			23	20	6		
Recommended by family members or friends	11	6			16	1			14	2	1			9	7	1		
Others	6	1			7	0			2	5	0			5	0	2		
Willingness to continue vaccinating																		
Yes	68	35	4.392	.111	100	3	1.854	.388	60	38	5	3.004	.530	53	40	10	4.868	.301
No	4	7			10	1			7	3	1			2	7	2		
Depends	18	15			32	1			17	12	4			16	13	4		
The reason you will not to continue to vaccinate (multiple choice)																		
High cost	20	21	3.709	.054	38	3	1.258	.262	28	12	1	3.555	.169	25	13	3	3.712	.156
Limited effect	29	21	0.332	.565	49	1	0.037	.847	26	21	3	1.165	.558	21	25	4	2.767	.251
Side effects and poor safety	15	14	1.374	.241	27	2	2.857	.240	14	12	3	1.448	.485	12	13	4	0.781	.677
Limited vaccination sites	45	50	0.269	.604	72	4	1.786	.181	40	29	7	2.095	.351	42	26	8	3.281	.194
Others	4	4	0.449	.503	7	1	3.470	.173	5	3	0	0.623	.732	1	6	1	4.687	.096
The reason you did not vaccinate (Q20) BS‐CC multiple choice)																		
High cost	17	35	1.848	.174	51	1	2.173	.140	37	11	4	0.084	.959	26	18	8	2.069	.356
In good health. The influenza is not a serious illness	74	139	6.083	.014	196	17	2.207	.137	157	37	19	4.387	.112	101	94	18	1.798	.407
Side effects and poor safety	35	47	0.044	.834	74	8	2.054	.152	58	20	4	0.983	.612	37	31	14	3.974	.137
Limited effect	16	22	0.004	.950	34	4	1.234	.267	27	10	1	1.532	.465	20	10	8	6.736	.034
Unknown vaccination sites	106	147	0.013	.909	244	9	4.274	.039	176	65	12	6.835	.033	119	103	31	1.143	.565
No time	62	81	0.227	.634	136	7	0.386	.534	108	26	9	1.077	.584	61	64	18	1.406	.495
Never heard of it	83	106	0.573	.449	184	5	5.231	.022	128	49	12	3.675	.159	94	77	18	1.098	.578
Vaccine shortage	7	22	3.812	.051	25	4	2.487	.115	24	5	0	2.729	.256	8	16	5	4.690	.096
Contraindications	4	4	0.234	.628	7	1	2.745	.207	7	0	0	2.373	.305	1	6	1	4.061	.131
Vaccination sites is far	13	34	4.053	.044	44	3	0.014	.907	35	12	0	3.912	.141	24	20	3	1.112	.574
Others	8	18	1.310	.252	23	3	1.195	.274	20	4	2	0.542	.763	13	9	4	0.992	.609
Do you intend to receive influenza vaccinate in the near future?																		
Yes	43	46	3.771	.152	83	6	1.361	.506	46	39	4	32.506	<.001	44	38	7	3.400	.493
No	137	220			333	24			272	59	26			157	156	44		
It depends	98	124			212	10			164	43	15			111	90	21		

### 3.5 Multivariate analysis

Then, we conducted a multivariate analysis to identify which factors affect the outcome of vaccination. The results are all shown in Table 8. The table affects factors including sex, age, education, major, income, and three single‐choice questions about “Have you ever heard of the influenza vaccine?,” “Do you think the influenza vaccine can protect you from getting influenza?,” “How necessary do you think vaccinating every year is?,” “How safe do you think the influenza vaccine is?,” and “Do you worry about getting influenza?.” The sex and age are two significant influence factors (*p* < .01). Heard of the influenza vaccine (*p* = .014) and “How necessary do you think vaccinating every year is?” (*p* sig < 0.001). As a result, the significant results may affect the vaccination. Other results were not significant difference.

**Table 8:** Binary logistic regression analysis of the impact of various factors on the behavior of being vaccinated with the influenza vaccine within the past 4 year.

		*β*	SE	Wald	*df*	Sig	Exp (*β*)	95% CI for exp (*β*)	Upper
								Lower	
Sex									
	Male	0.772	0.214	13.036	1	<0.001	2.163	1.423	3.289
	Female						1.000		
Age									
	<20	0.807	0.210	14.750	1	<0.001	2.242	1.485	3.385
	≥20						1.000		
Education									
	Undergraduate students	0.125	0.518	0.059	1	0.809	1.134	0.411	3.129
	Master students						1.000		
Major				1.769	2	0.413			
	Medicine	−0.102	0.400	0.064	1	0.800	0.903	0.412	1.979
	Engineering	0.212	0.419	0.256	1	0.613	1.236	0.544	2.807
	Science and others						1.000		
Income				0.053	2	0.974			
	<3000	0.031	0.334	0.008	1	0.927	1.031	0.536	1.985
	3000−10,000	−0.017	0.336	0.003	1	0.959	0.983	0.509	1.899
	>10,000						1.000		
Have you ever heard of the influenza vaccine?									
	Yes	0.976	0.399	5.998	1	0.014	2.655	1.215	5.801
	No						1.000		
Do you think the influenza vaccine can protect you from getting influenza?				4.665	2	0.097			
	Yes	0.561	0.429	1.715	1	0.190	1.753	0.757	4.060
	No	–0.397	0.665	0.355	1	0.551	0.673	0.183	2.478
	Do not know						1.000		
How necessary do you think vaccinating every year is?				19.744	2	<0.001			
	Necessary	1.373	0.323	18.123	1	<0.001	3.947	2.098	7.427
	Not necessary	0.843	0.338	6.242	1	0.012	2.324	1.199	4.505
	Do not know						1.000		
How safe do you think the influenza vaccine is?				2.568	2	0.277			
	It is safe and with no side effects.	0.068	0.944	0.005	1	0.943	1.070	0.168	6.804
	It is basically safe and with some side effects.	−0.335	0.919	0.133	1	0.715	0.715	0.118	4.330
	It is not safe and with obvious side effects.						1.000		
Do you worry about getting influenza?				3.191	2	0.203			
	Very worried	0.534	0.321	2.766	1	0.096	1.706	0.909	3.202
	Worried	0.129	0.257	0.250	1	0.617	1.137	0.687	1.883
	Not worried						1.000		

## 4 DISCUSSION

As we know, this study is one of the first surveys of the college students' KAP toward influenza vaccination during the COVID‐19 pandemic in Southern China. We conducted a cross‐sectional survey during January 2021, and get 815 valid questionaries. As we know, no large‐scale surveys of the COVID‐19 vaccination of children about college students. The innovation of this survey is that we conduct a survey among college students using the questionnaire survey and collection analysis, and multivariate analysis had been conducted which can explore the factors affecting vaccination.

College students were not generally grasping the knowledge of the influenza vaccination well. Only a few participants knew the right season of vaccination, and one‐third of participants recognized influenza vaccination as unnecessary. However, the awareness rate still improved compared to the research of Yu et al.^2^ in 2018; the report showed that only 6.47% of participants were clearly aware of the vaccination timeframe, and nearly half (45.51%) of the participants thought that influenza vaccination was not necessary. It may result from the higher education levels of participants in this study (bachelor or master). Higher education levels may relate to better influenza knowledge levels.^18^ What's more, due to the mass media reporting the knowledge about COVID‐19 and influenza during the COVID‐19 outbreak, participants may be educated by the mass media.^19^

In terms of attitude toward influenza vaccination, participants showed high recognition. The majority of participants believe that the influenza vaccine was safe and effective, and will advise family and friends to be vaccinated. However, the influenza vaccination rate is 18%. Although it is slightly higher than the previous study in China,^19^, ^20^, ^21^, ^22^ but still lower than the developed regions such as European countries (35.6%), and America (61.2%).^23^, ^24^ As a result, the vaccination in China should be paid more attention.

The economic status may influence the vaccination rate. From 2011 to 2015, after Beijing provided funding for influenza vaccination, the vaccination rate for the elderly increased to nearly 50%.^25^ The financial support provided by the government for influenza vaccination for susceptible people has an important impact on the increasing rate of influenza vaccination. National policy support for influenza vaccines can improve vaccination rates.

In our study, the main reason for not getting vaccinated was not knowing the vaccination sites (31%) and the second important reason was that the participants believed they were in good health and influenza was not a serious illness (26.1%). The research of Yu et al.^2^ and Jiang et al.^19^ showed similar results, either. So, it was important to improve the convenience of vaccination and people's recognition of influenza. Similar to other studies, doctors' recommendation is the most important approaches that led to being vaccinated in our research.^26^, ^27^, ^28^, ^29^ It's crucial that healthcare workers (HCW) provide more vaccination recommendations. Therefore, it is necessary to improve the level of vaccine knowledge of HCW, carry out vaccine knowledge training for hospital doctors, and enhance their confidence in the vaccine, so that realization of the health intervention role of HCW.

Social media also plays an important role in vaccination, it may influence people's adherence to vaccination guidelines.^30^ To let people know about the vaccination sites and other related information, we can post the vaccination‐related information on social media and guarantee the authenticity and rationality of the information.^31^

Then, health education is carried out in schools, through lectures or other activities to publicize the relevant knowledge and immune effect of the influenza vaccines. Combined with the above effective methods, the level of influenza vaccination can be further improved.

However, our data was limited in the college students and limited samples, and second, our study used the convenience sampling so that some bias can't be diminished.

All in all, during the COVID‐19 epidemic, we should further improve the KAP toward influenza vaccination, and targeted intervention measures for the obstacles to prevent our global health in the future.

## AUTHOR CONTRIBUTIONS

*Conceptualization*: Di Wu and Pengzhe Qin. *Data curation*: Pan Liu, Jie Long, and Tiantian Wu. *Formal analysis*: Xuejiao Chen and Weiquan Lin. *Methodology*: Qun Liu and Dedong Wang. *Writing*—*original draft*: Di Wu and Yuexue Mai. *Writing*—*review and editing*: Xuejiao Chen, Zhoubin Zhang, Di Wu, and Pengzhe Qin.

## CONFLICT OF INTEREST STATEMENT

The authors declare no conflict of interest.

## Data Availability

Data is available on request from the authors.
